# Screening and brief counseling among young adults: comparison of college students, community college students, and persons not in school

**DOI:** 10.1186/1940-0640-10-S2-P7

**Published:** 2015-09-24

**Authors:** Ralph Hingson, Wenxing Zha, Aaron White, Bruce Simons-Morton

**Affiliations:** 1 Division of Epidemiology and Prevention Research, National Institute on Alcohol Abuse and Alcoholism, Bethesda, MD, USA; 2 Division of Intramural Population Health Research, National Institute of Child Health & Human Development, USA

## Background

The objectives are to explore proportions of 4-year and community college freshmen and same-age, non-college peers who 1) saw a physician in the past year; 2) were asked and advised about substance use and other behavioral health risks; and 3) were asked to reduce or stop substance use and change other risky behaviors.

## Material and methods

A national probability sample of 10th graders interviewed in 2009 (N= 2,519) was re-interviewed [N=2,140 (84%)] the first year after high school. Questions addressed physician contact and whether physicians asked and counseled subjects about substance use and other behavioral health risks. Chi square tests assessed significance of response variation between groups.

## Results

Of respondents, 42% were enrolled in 4-year, 25% in community colleges, and 33% were not enrolled. Students in 4-year and community colleges were more likely than others to have seen a physician in the past year (75%, 73%, 65%). Of those seeing a physician, similar proportions (75%-82%) in each group were asked about their drinking, smoking, and drug use. Students in 4-year and community colleges were less likely to be advised about health risks linked to drinking (45%, 46%, vs. 53%), smoking (45%, 47%, vs. 57%), and using drugs (44%, 46%, vs. 53%). Of 4-year and community college students and those not enrolled, 15%, 19%, and 28%, respectively, were advised to reduce or stop drinking; 14%, 20%, and 30% smoking; and 14%, 20%, and 26% drug use. Higher percentages were encouraged to exercise, improve their diet, and avoid pregnancy and sexually transmitted diseases.

## Conclusions

More physicians should ask and advise emerging adults about substance use risks and to reduce or stop substance use. Advice to reduce or stop drinking is particularly needed, as alcohol is the most used substance in that group and often used in hazardous ways, especially by college students.

## References

[B1] U.S. Department of Health and Human Services The Surgeon General's Call to Action to Prevent and Reduce Underage Drinking 2007 Washington, DC: U.S. Department of Health and Human Services, Office of the Surgeon General 20669519

[B2] Moyer VA Preventive Services Task Force Screening and behavioral counseling interventions in primary care to reduce alcohol misuse: U.S. preventive services task force recommendation statement Ann Intern Med 2013 159 3 210 8 23698791 10.7326/0003-4819-159-3-201308060-00652

[B3] Scott-Sheldon LA Carey KB Elliott JC Garey L Carey MP Efficacy of alcohol interventions for first-year college students: a meta-analytic review of randomized controlled trials J Consult Clin Psychol 2014 82 2 177 88 24447002 10.1037/a0035192 PMC3987817

[B4] Tanner-Smith EE Lipsey MW Brief alcohol interventions for adolescents and young adults: a systematic review and meta-analysis J Subst Abuse Treat 2015 51 1 18 25300577 10.1016/j.jsat.2014.09.001 PMC4346408

[B5] Hingson RW Zha W Iannotti RJ Simons-Morton B Physician advice to adolescents about drinking and other health behaviors Pediatrics 2013 131 2 249 57 10.1542/peds.2012-1496 23359580 PMC4074674

[B6] Hingson R Zha W Magnitude of and trends in alcohol-related mortality and morbidity among U.S. College Students Ages 18-24, 1998-2005 J Stud Alcohol Drugs 2009 Suppl. 16 12 20 10.15288/jsads.2009.s16.12 PMC2701090 19538908

